# Tunable mechanical properties and phase transitions in nanoconfined polyzwitterionic UCST hydrogels†

**DOI:** 10.1039/d5sm00317b

**Published:** 2025-04-15

**Authors:** Sebastian Loescher, Chen Liang, Remi Plamont, Josef Breu, Olli Ikkala, Hang Zhang

**Affiliations:** a Department of Applied Physics, Aalto University P.O. Box 15100 02150 Espoo Finland olli.ikkala@aalto.fi hang.zhang@aalto.fi; b Bavarian Polymer Institute and Department of Chemistry, University of Bayreuth, Universitätsstrasse 30 95440 Bayreuth Germany josef.breu@uni-bayreuth.de; c Department of Bioproducts and Biosystems, School of Chemical Engineering, Aalto University P.O. Box 16100 02150 Espoo Finland

## Abstract

Stimuli-responsive hydrogels with thermal phase transitions serve as pivotal components in advancing biomedical and soft robotics applications. In contrast to widely studied LCST-type thermo-responsive hydrogels, UCST-type hydrogels provide reverse thermo-responses. However, conventional UCST-type hydrogels suffer from weak mechanical properties and fixed phase transition kinetics. Here, we present polyzwitterionic UCST-type hydrogels under coplanar nanoconfinement by large aspect ratio hectorite nanosheets. The nanoconfinement significantly enhances the strength and stiffness of the hydrogels. In addition, the nanosheets serve as kinetic barriers for water diffusion. This regulates the swelling and shrinking kinetics of the polyzwitterionic hydrogels and thus allows for tunable phase transitions dependent on the thermal history of the hydrogels. Furthermore, we demonstrate that the incorporation of gold nanoparticles allows precise control of the optical properties of the hydrogel through photothermal means. These findings pave the way for engineering both the mechanical and thermoresponsive properties in polyzwitterionic hydrogels, thus broadening their applications in smart soft materials.

## Abstract 2

Polyzwitterionic UCST hydrogels confined within hectorite nanostructures exhibit enhanced strength and tunable phase transitions dependent on thermal history, broadening their applications in smart soft materials.

## Graphical abstract

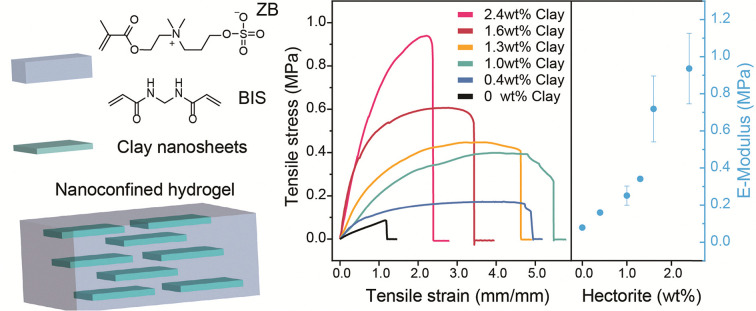

## Introduction

Hydrogels represent a distinctive category of materials known for their capacity to absorb and retain significant quantities of water within a three-dimensional, crosslinked polymeric network.^1^ They are frequently utilized to mimic the functions and stimuli-responsive behaviors of soft biological tissues, including structural support, responsive behaviors,^2^ molecular crowding when high concentrations of macromolecules in a solution alter their properties,^3–5^ and dynamic swelling/shrinking transitions,^6,7^ making them invaluable in biomedical engineering, soft robotics, and smart material design. Among these properties, thermoresponsive phase transitions,^8^ such as the volume phase transitions (VPT) of lower critical solution temperature (LCST)-type polymers (*e.g.*, poly(*N*-isopropylacrylamide), pNIPAM),^9,10^ have been extensively studied for applications like drug delivery, soft robotics,^11^ and smart materials.^12^

In contrast, hydrogels based on upper critical solution temperature (UCST)-type polymers remain underexplored despite their unique reverse phase behavior,^13–20^ which forms a miscibility gap below the critical temperature. The phase transitions in UCST-type hydrogels are highly sensitive to ionic comonomer impurities,^13^ H-bonding competitors,^14^ or ionic strength of the surrounding aqueous media.^15^ Recently, Gong and co-workers have reported on self-healing polyampholyte hydrogels that undergo UCST type transitions with negligible volume change and adaptive temperature response, which were exploited as memory-type response with forgetting ability.^16,17^ While polyzwitterionic and polyampholyte UCST-type hydrogels have attracted recent attention due to their high swellability, self-healing capabilities, and biocompatibility,^18–20^ their practical utility is hindered by two critical limitations: (1) inherently weak mechanical properties (*e.g.*, low stiffness and strength) and (2) transition kinetics that are predefined by their chemical composition, leaving little room for tunability. Previous studies to achieve mechanical reinforcement^21–23^ in polyzwitterionic hydrogels or to tune the UCST phase transitions^24,25^ typically lack either of the two properties.

In this study, we overcome these challenges by incorporating synthetic high aspect ratio (>20 000) hectorite nanosheets ([Na_0.48_]^inter^ [Mg_2.57_ Li_0.47_]^oct^ [Si_4_]^tet^ O_10_ F_2_)^26^ into a poly(sulfabetaine) (ZB) hydrogel network loosely crosslinked with *N,N*′-methylenebis(acrylamide) (MBAA). This strategy introduces a nanoconfinement effect^27^*via* hectorite nanosheets to reinforce the hydrogel without sacrificing the UCST-behaviour. Crucially, the nanosheets act as two-dimensional barriers for water diffusion, enabling facile control over the equilibration kinetics of the phase transition by the clay concentration. Furthermore, addition of gold nanoparticles allows control over the transparency and phase transition behaviours of the hydrogels by photothermal means. The nanoconfinement strategy addresses the long-standing trade-off between mechanical strength and thermoresponsiveness in UCST hydrogels and establishes a general route in tuning UCST hydrogels towards programmable smart soft materials.

## Results and discussion

**Fig. 1:**
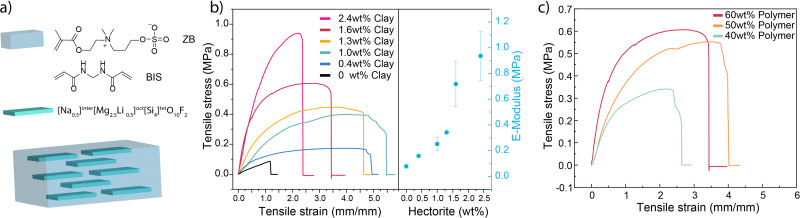
Zwitterionic hydrogels containing high aspect ratio hectorite clays. (a) Chemical structures of the ZB monomer, chemical crosslinker and hectorite nanosheet. (b) Mechanical properties of hydrogels with 60 wt% of the ZB content, 0.1 g L^−1^ BIS and various wt% of hectorite. (c) Mechanical properties of various polymer wt% and 1.6 wt% clays.

The hydrogels were prepared by photoinitiated radical polymerization of the monomer in the presence of hectorite nanosheets and a small amount of crosslinkers (see Fig. 1a). The synthesized ZB monomer was characterized by ^1^H NMR (Fig. S1, ESI†). The nanosheets are uniformly delaminated as 1 nm thin monolayers in water due to uniform charge density,^28^ where the separation can be adjusted by the concentration of the nanosheets. The high aspect ratio of the hectorite allows the spontaneous liquid crystallinity and facile orientation of the hectorite nanosheets along the direction of mild flow during solution injection into thin glass moulds (see the Experimental section).

The pure ZB polyzwitterionic hydrogels show relatively weak mechanical properties without the addition of hectorite nanosheets, similar to conventional polyzwitterionic hydrogels. Even at a polymer content of 60 wt%, the pure polyzwitterionic hydrogel exhibits a Young's modulus of only 100 kPa, a fracture strength of less than 100 kPa, and 100% elongation at break (see Fig. 1b), showing their fragile nature. In contrast, the mechanical properties increased significantly upon the incorporation of nanosheets. Here, we denote the hydrogels as ZB_*m*_-clay_*n*_, where *m* and *n* indicate the weight percentage of ZB and hectorite in the as-prepared hydrogel, respectively. At a hectorite concentration of around 1.5 wt%, the modulus of the hydrogel undergoes a significant increase, which demonstrates the nanoconfinement effect as the separation between the equidistant nanosheets approaches 100 nm.^27^ Our previous study^27^ has shown that the co-planar spacing between synthetic clay nanosheets acts as nanoconfinement that approaches the dimension of polymer chains, thus enhancing the stiffness and strength of the hydrogel. The Young's modulus of the ZB-hectorite hydrogel can reach up to 0.9 MPa with 2.4 wt% nanosheets, *i.e.*, a ten-fold increase compared to ZB hydrogels without hectorite. There exists a strong increase in the modulus at a hectorite concentration of around 1.5 wt%, reminiscent of that reported earlier in the nanoconfined hydrogels.^27^ Similarly, the elongation at break increases to around 500% for hectorite contents ranging from 0.4% to 1.3%. The ultimate tensile strength of the ZB-hectorite hydrogels also underwent a dramatic improvement to above 0.9 MPa at a hectorite concentration of 2.4 wt%, a ten-fold increase compared to ZB hydrogels without hectorite. The stretchability of the hydrogel decreases at clay concentrations above 1.3 wt%, possibly due to limited chain dynamics at elevated clay concentrations. At a constant hectorite content of 1.6 wt%, the mechanical properties decrease with lower polymer contents (see Fig. 1c), which are still significantly higher than pure ZB hydrogels without hectorite. These results demonstrate that the addition of hectorite nanosheets can serve as an effective approach to reinforce the mechanical properties of polyzwitterionic hydrogels.

**Fig. 2:**
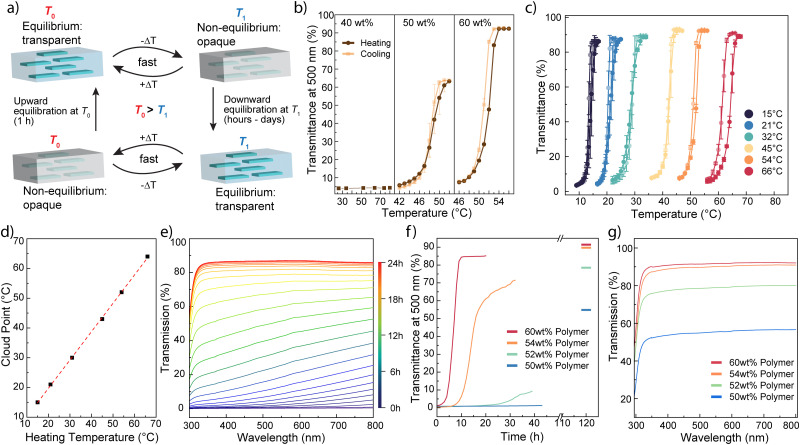
Characterization of phase transitions in hydrogels. (a) Overview of the fast phase transition response between non-equilibrium and equilibrium states. Downward equilibration indicates equilibration from a higher temperature *T*_0_ to a lower temperature *T*_1_, while upward equilibration indicates equilibration from a lower temperature to a higher temperature. The fast change of temperature around equilibration temperature allows the swift response without affecting the UCST. (b) Optical response of hydrogels equilibrated at 53 °C for different polymer wt%. The data are mean values with standard deviations as error bars from 3 measurements. (c) Upon equilibration for 1 h at the indicated temperatures, the cloud point of the hydrogel adjusts itself to just slightly below the equilibration temperature. The polymer content is 60 wt%. The data are mean values with standard deviations as error bars from 3 measurements. (d) The resulting cloud points correlate well with the equilibration temperature. (e) Exemplary kinetic course of downward RT equilibration of a 60 wt% hydrogel after 15 min at 60 °C. (f) The recovery after 15 min 60 °C depends on the polymer wt% of the hydrogel. (g) Hydrogels with a higher polymer wt% show higher optical transparency.

Besides enhanced mechanical properties, the ZB-hectorite hydrogels show a thermal history-dependent tunable phase transition temperature similar to recently reported polyampholyte hydrogels.^29^ The ZB-hectorite hydrogel shows a UCST-type transition just slightly below its thermal equilibration temperature, defined as the temperature at which the gel has been incubated for an extended period of time (>1 hour). Through equilibration at a new temperature, a new transition temperature is acquired (Fig. 2a). The kinetics of upward (heating) and downward (cooling) equilibrations differ significantly with the latter being much slower.

The phase transitions in the ZB-hectorite hydrogels are characterized using UV-Vis transmittance measurements, as shown in Fig. 2. During phase transitions or equilibrations, the size of the hydrogel remained unchanged. Opaqueness in hydrogels arises below the UCST due to the emergence of scattering centres at phase boundaries as a result of the conformational change in the polymers and subsequent formation of polymer-rich and polymer-poor phases.^30^ Here, the phase transition temperature is defined as the point at which a fifty percent change in optical transparency at 500 nm is observed. ZB-type polymers are known to exhibit high UCST even in the presence of significant amounts of electrolytes.^31,32^ In ZB-hectorite hydrogels containing only 40 wt% of ZB that are equilibrated at 54 °C, the hydrogels remain opaque even up to a temperature of 80 °C as shown in Fig. 2b. In contrast, hydrogels with higher ZB contents, such as 50 wt% and 60 wt%, displayed optical transparency changes during phase transitions. This difference is due to the fact that UCST strongly depends on the concentration of the polyzwitterionic polymers,^33^ which resulted in a UCST above 80 °C for the 40 wt% ZB hydrogel.

Notably, the UCST phase transition temperatures in the ZB-hectorite hydrogels show strong dependence on the thermal history, *i.e.*, a memory effect. For instance, the phase transition temperature of ZB_60_-clay_1.6_ hydrogels shows a strong linear dependence of the temperature at which it was equilibrated for 1 h (upward from lower temperatures), as shown in Fig. 2c and d, in the broad range between 15 and 66 °C. This behaviour is reminiscent to previous report on polyampholyte hydrogels,^17^ which undergoes structure frustration with an entrapped water phase. When the ZB_60_-clay_1.6_ hydrogel was heated for 1 hour to 45 °C, it equilibrated and established a new UCST response at around 45 °C. If the temperature was quickly decreased below the new UCST temperature, the hydrogel undergoes a phase transition, resulting in a decrease of optical transparency. The newly acquired UCST-type transition of the ZB-hectorite hydrogel remains unchanged upon fast temperature changes, such as rapid heating (12 °C min^−1^) and cooling (6 °C min^−1^) cycles around the transition shown in Fig. 2c. The same hydrogel can undergo many cycles of equilibrations at various temperatures, where they can adopt the new temperature as the new UCST-type transition. To the best of our knowledge, this effect has not yet been reported for charge balanced polyzwitterionic hydrogel nanocomposites.

The hydrogels equilibrated at elevated temperatures undergo much slower downward equilibration and will remain optically opaque for an extended period of time. Already after 15 minutes of upward equilibration, the hydrogels show long opaque lifetimes upon downward equilibration as it returns to its transparent state (Fig. 2e). Interestingly, the lifetime also highly depends on the polymer content of the hydrogels, with lower contents resulting in substantially longer opaque lifetimes (Fig. 2f). Similarly, with the phase transitions, lower polymer wt% also shows lower optical transparency in a fully equilibrated state, underlining again the high UCST transition temperature of ZB polymers.

**Fig. 3:**
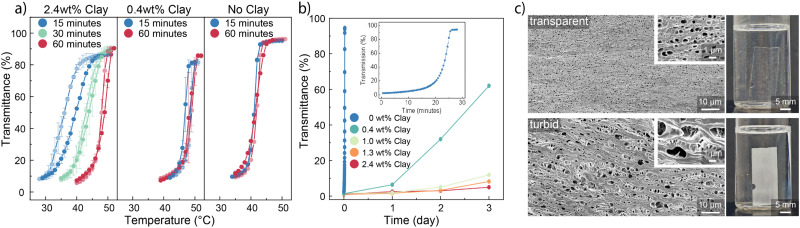
Influence of large aspect ratio hectorite clay on equilibration kinetics. (a) The presence of hectorite clay leads to significantly slower equilibration kinetics during upward equilibration at 50 °C. In the absence of clay, equilibration is fast but the transition temperature is around 10 °C lower than the heating temperature. The data are mean values with standard deviations as error bars from 3 measurements. (b) In the absence of clay, the downward equilibration kinetics is fast, whereas small amounts of clay lead to much slower kinetics in the range of days. (c) Scanning electron micrographs and optical photographs of transparent and turbid hydrogels reveal the size difference of polymer- and water-rich domains leading to strong scattering in the case of the turbid gel.

Fig. 3 demonstrates the influence of the hectorite concentration on the equilibration kinetics of our hydrogels. The equilibration time needed for the hydrogel to acquire a new UCST is considerably extended with increasing hectorite concentrations (Fig. 3a). For the ZB_60_-clay_2.4_ hydrogel, it takes about 1 h of heating to 50 °C for fully adapting the UCST transition temperature to 50 °C. In contrast, the ZB_60_-clay_0.4_ hydrogel requires only 15 minutes. This is due to the fact that the clay nanosheets act as diffusion barriers for water, which slows down the equilibration process. With increasing concentration of clay nanosheets, longer time is required for water to fully equilibrate within the hydrogel.^34^ As a control, the hydrogel containing no clay equilibrates within 15 minutes; however, the final equilibrated phase transition temperature is around 10 °C lower than the equilibration temperature. This difference in the equilibrated transition temperature could be attributed to the interactions between the zwitterionic polymers and the anionic clays, which alters the UCST behaviours. Therefore, the presence of the clay both influences the equilibration kinetics and the final transition temperature of the hydrogel.

For the downward equilibration, where the hydrogel has been pre-equilibrated at 50 °C for 1 h, the kinetic trends are similar to the upward one shown in Fig. 3b. The ZB hydrogel without clay equilibrates swiftly within 25 minutes whereas with increasing hectorite content, the kinetics slow down significantly. Already, for 0.4 wt% hectorite content, the equilibration to room temperature takes more than 3 days, whereas hydrogels with even higher hectorite contents are still completely turbid after 3 days, indicating a much more slowed-down process in the presence of hectorite nanosheets.

The microstructures of the hydrogel ZB_60_-Clay_1.6_ are characterized by scanning electron microscopy using freeze-dried samples as shown in Fig. 3c. The turbid and kinetically trapped hydrogel displayed larger and more widely distributed pores in the polymer matrix. In contrast, the fully equilibrated, transparent hydrogels exhibited a more uniform microstructure, characterized by much smaller and uniform distribution of pores. The larger domain features observed in the turbid hydrogel act as Mie scattering centres, resulting in the pronounced opaqueness of the hydrogels.

**Fig. 4:**
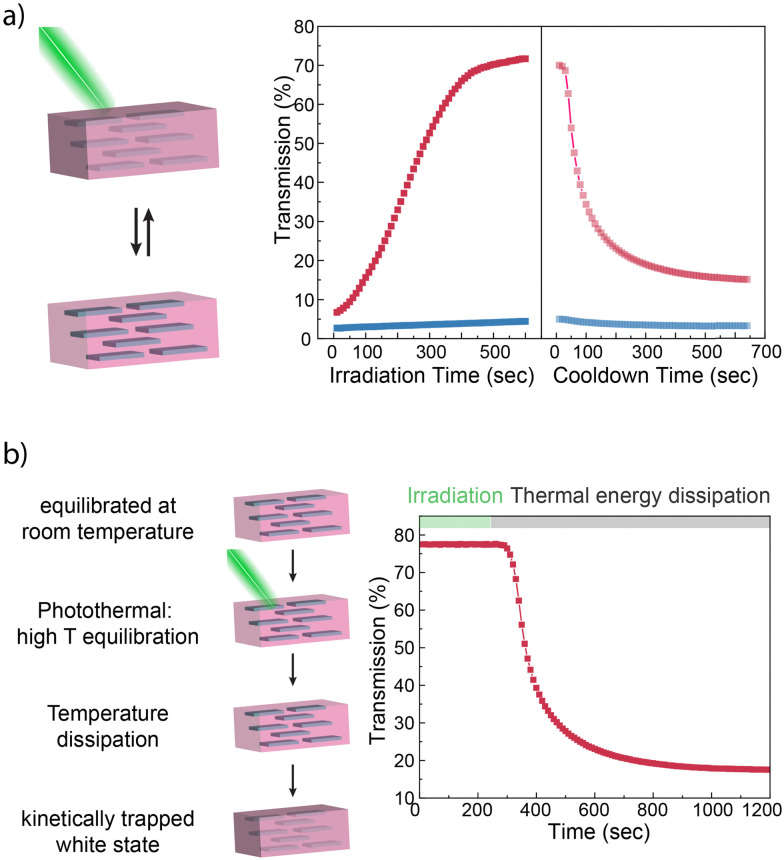
Photothermally induced phase transition in AuNP containing ZB-hectorite hydrogels. (a) Upon irradiation, a hydrogel preconditioned at 50 °C undergoes a turbid-transparent transition through photothermal heat build-up. In the absence of light irradiation (red), the hydrogel returns quickly back to its opaque state. In the absence of AuNPs, no phase transition occurred (blue). (b) A room temperature equilibrated and transparent hydrogel undergoes no visible optical change upon irradiation. After the light source is removed, the hydrogel quickly turns opaque due to thermal conditioning.

To allow further control of the hydrogel turbidity using light, we introduced photothermal agents into our hydrogels to enable controlled phase transition through an external light source, as shown in Fig. 4. We selected cationic gold nanoparticles (AuNPs) with a diameter of 13 nm for this purpose and prepared by a controlled seeded-growth method^35^ and subsequent ligand exchange using (11-mercaptoundecyl)-*N*,*N*,*N*-trimethylammonium bromide (MUTAB).^36^ The cationic MUTAB@AuNPs were stable in the dispersion of hectorite nanosheets (see Fig. S2, ESI†). The electrostatic interactions between cationic AuNPs and the anionic clay nanosheets as well as the polysulfabetaine network^37^ prevent the leakage of AuNPs even after multiple heating–cooling cycles of these gels. Indeed, no leakage was observed after multiple heating–cooling cycles. The addition of AuNPs did not affect the phase transition behaviours in the ZB-hectorite hydrogels (Fig. S3, ESI†).

When irradiated at the maximum of the absorption of the AuNPs with a green LED light source (525 nm and 50 mW cm^−2^), the temperature of the hydrogel pre-conditioned at 50 °C (*i.e.*, with an UCST of 50 °C) increased through photothermal heat conversion, leading to a phase transition in the gel. The gel became transparent within a few minutes of green light irradiation. Upon removal of the light source, the gel quickly reverted below its UCST temperature, and turbidity was restored. In contrast, a hydrogel lacking AuNPs displayed no phase transition, and NIR camera measurements confirmed that its temperature changed only by a few degrees during irradiation. In addition, a room temperature-equilibrated and transparent gel was irradiated with the green LED for 5 minutes, during which its optical properties remained unchanged, *i.e.*, in a transparent state. However, after switching off the light source, the hydrogel retained its transparency for a brief period before swiftly becoming turbid due to the newly acquired higher phase transition temperature. The combination use of temperature and light thus allows further temporal control and programmability of the turbidity of the hydrogel.

## Conclusions

In summary, our study has elucidated the impact of high-aspect-ratio nanosheets on the mechanical properties and the equilibration kinetics of nanoconfined polyzwitterionic hydrogels. The addition of hectorite nanosheets significantly improved the mechanical properties of the hydrogel *via* the nanoconfinement effect. These hydrogels exhibit a UCST-type phase transition under kinetic control, which is contingent on the thermal history of the gel. Importantly, we can precisely tune the UCST temperature through upward thermal equilibration within an hour. Conversely, when undergoing downward equilibration, the hydrogels exhibit significantly longer lifetimes of the UCST, extending over several days, depending on the initial equilibration temperature. The presence of hectorite nanosheets prolongs the equilibration kinetics, suggesting their role as a barrier to slow down water diffusion within the hydrogel. Furthermore, the incorporation of gold nanoparticles enables photothermal phase transitions *via* an external light source. By irradiating the hydrogels with light, they can be encoded with a new state, transitioning through two distinct transparency states, ultimately showing turbidity. Our findings provide new means for fine-tuning UCST behaviours in hydrogels that will lead to an enhanced spatiotemporal control for a new generation of adaptive and smart materials.

## Experimental

### Materials

All chemicals were used without prior purification. 2-(Dimethylamino)ethyl methacrylate, 1,3-propanediol cyclic sulfate, hexadecyltrimethylammonium bromide, *N*,*N*′-methylenebisacrylamide (BIS), 2-hydroxy-4′-(2-hydroxyethoxy)-2-methylpropiophenone (photo initiator), gold(iii) chloride trihydrate, (11-mercaptoundecyl)-*N*,*N*,*N*-trimethylammonium bromide, sodium borohydride and acetonitrile were purchased from Sigma Aldrich. The large aspect ratio hectorite nanoclay was synthesized according to a previously reported procedure.^22,23^

### Synthesis of the ZB monomer

The ZB monomer was synthesized according to literature reports.^31^ In brief, 2-(dimethylamino)ethyl methacrylate (23.3 g, 25 mL, 148 mmol) and 1,3-propanediol cyclic sulfate (20.5 g, 148 mmol) were dissolved in acetonitrile (400 mL). Under stirring, few drops of nitrobenzene were added and the mixture stirred over night at 50 °C. The final product was washed with acetone and dried using a rotary evaporator. The monomer was obtained as white powder in 80% yields. ^1^H NMR (400 MHz, D_2_O) *δ* = 6.23 (t, *J* = 1.1, 1H), 5.85 (p, *J* = 1.5, 1H), 4.74–4.68 (m, 2H), 4.22 (t, *J* = 5.7, 2H), 3.92–3.86 (m, 2H), 3.70–3.61 (m, 2H), 3.29 (s, 6H), 2.38–2.26 (m, 2H), 2.01 (t, *J* = 1.3, 3H).

### Preparation of UCST-type hydrogels

The hydrogels were prepared in custom-made molds consisting of a polypropylene sheet and a glass sheet separated by polymeric films of defined thickness (0.5 mm). A solution consisting of the hectorite clay nanosheets ([Na_0.48_]^inter^ [Mg_2.57_ Li_0.47_]^oct^ [Si_4_]^tet^ O_10_F_2_), the ZB monomer (3-((2-(methacryloyloxy)ethyl)dimethylammonio)propyl sulfate) powder in the indicated ratios and the photoinitiator (1 mg mL^−1^) and crosslinker BIS (0.1 mg mL^−1^) were mixed thoroughly until a clear solution was obtained. The solutions were exposed to a nitrogen atmosphere and transferred into the previously described molds within a glovebox. The hydrogels were polymerized at 50 °C in the presence of UV light (365 nm) for 3 h. After polymerization, the hydrogels were removed from the mold and stored in deionized water at room temperature.

### Preparation of AuNPs

To synthesize gold nanoparticles with an average diameter of 13 nm, the seeded growth method in the presence of tannic acid was used.^28^ All solutions were freshly prepared before the reaction. Briefly, 150 mL of 2.2 mM aqueous solution of trisodium citrate dihydrate was mixed with 0.1 mL of 2.5 mM tannic acid and 1 mL of 150 mM potassium carbonate in a 250 mL three-neck flask. The solution was heated to 70 °C in an oil bath. Subsequently, 1 mL of 25 mM HAuCl_4_·3H_2_O solution was quickly injected under vigorous stirring, and the solution was kept at 70 °C for another 5 min. This led to preparing gold seeds with an average diameter of 3.5 nm. Directly after the formation of gold seeds, 55 mL of this solution was extracted, and 55 mL of 2.2 mM citrate solution was added. After the temperature of the solution reached again 70 °C, 0.5 mL of 25 mM HAuCl_4_·3H_2_O solution was injected to initiate the growth of the seed. After 10 min, an identical injection was carried out. This growth procedure including extraction, the addition of citrate, and two injections of the gold precursor was repeated six times to obtain 13 nm AuNPs.

### Mechanical analysis of hydrogels

The mechanical properties of the hydrogels were measured using an Instron 5567 mechanical tester. The measurements were performed in controlled humidity to avoid drying of the hydrogels. E-Moduli were estimated using triplicate measurements.

### UV-VIS characterization of phase transitions

The optical responses of the hydrogels were measured using an Agilent Cary 5000 UV-Vis-NIR spectrophotometer with a temperature-controlled cell compartment. The extinction was measured against a reference sample in a dual beam set-up. The temperature was recorded with a temperature probe inside the cuvette. The hydrogels were immersed in MQ water in quartz cuvettes and either a full spectrum or a single wavelength (500 nm) was recorded. For the UCST-type measurements, the heating rate was 12 °C min^−1^, whereas the cooling rate was 7 °C min^−1^. The cloud point was defined as the half of the maximum transparency of the hydrogels. The optical recovery of hydrogels was measured in 0.5 h intervals at ambient temperature.

### SEM measurements

SEM measurements were conducted using a Sigma VP SEM (Zeiss, Germany) at an accelerating voltage of 3 kV. The cross-section of the hydrogel was prepared by freezing and cutting in liquid nitrogen, followed by freeze-drying for 24 h. The samples were sputtered with 4 nm Au–Pd before the measurement.

### Photothermally induced phase transitions

For the photothermally induced phase transitions, a Solis 525C lamp with a nominal maximum power of 3.1 W and a wavelength 525 nm was used. The real incident power on the hydrogel was estimated to be 50 mW cm^−2^. Perpendicular to the photothermal irradiation source, the transparency change of the hydrogel was measured as a single wavelength in continuous mode. The hydrogel was immersed in MQ inside a sealed cuvette.

## Author contributions

S. L. and H. Z. conceived the idea and designed the experiments. J. B., O. I. and H. Z. supervised the project. S. L. conducted the hydrogel-related experiments. C. L. performed the SEM characterization and analysis of the hydrogel. R. P. synthesized the gold nanoparticles. All authors contributed to the writing of the manuscript and conclusion.

## Data availability

The authors confirm that the data supporting the findings of this study are available within the main article and the ESI.† Additional supporting data, such as numerical data corresponding to the data presented in the figures, are available upon request to the corresponding authors.

## Conflicts of interest

The authors declare no conflicts of interest.

## Supplementary Material

SM-021-D5SM00317B-s001
