# Supplementary material for: Tunable mechanical properties and phase transitions in nanoconfined polyzwitterionic UCST hydrogels
Source: Soft Matter. 2025 Apr 15;21(20):4003–9. doi: 10.1039/d5sm00317b (PMC12038794; doi:10.1039/d5sm00317b)
Supplement: SM-021-D5SM00317B-s001 [file SM-021-D5SM00317B-s001.pdf]

Supplementary Information (SI) for Soft Matter.
This journal is © The Royal Society of Chemistry 2025

Supplementary Information

Tunable mechanical properties and phase transitions in nanoconfined polyzwitterionic UCST hydrogels

Sebastian Löscher,[a] Chen Liang,[a] Remi Plamont,[a] Josef Breu,*[b] Olli Ikkala,*[a] and Hang Zhang*[a,c]

a. Department of Applied Physics, Aalto University, P.O. Box 15100, 02150 Espoo, Finland.

b. Bavarian Polymer Institute and Department of Chemistry, University of Bayreuth, Universitätsstrasse 30, 95440 Bayreuth, Germany.

c. Department of Bioproducts and Biosystems, School of Chemical Engineering, Aalto University, P.O. Box 16100, 02150 Espoo, Finland.

Correspondence to josef.breu@uni-bayreuth.de, olli.ikkala@aalto.fi, and hang.zhang@aalto.fi

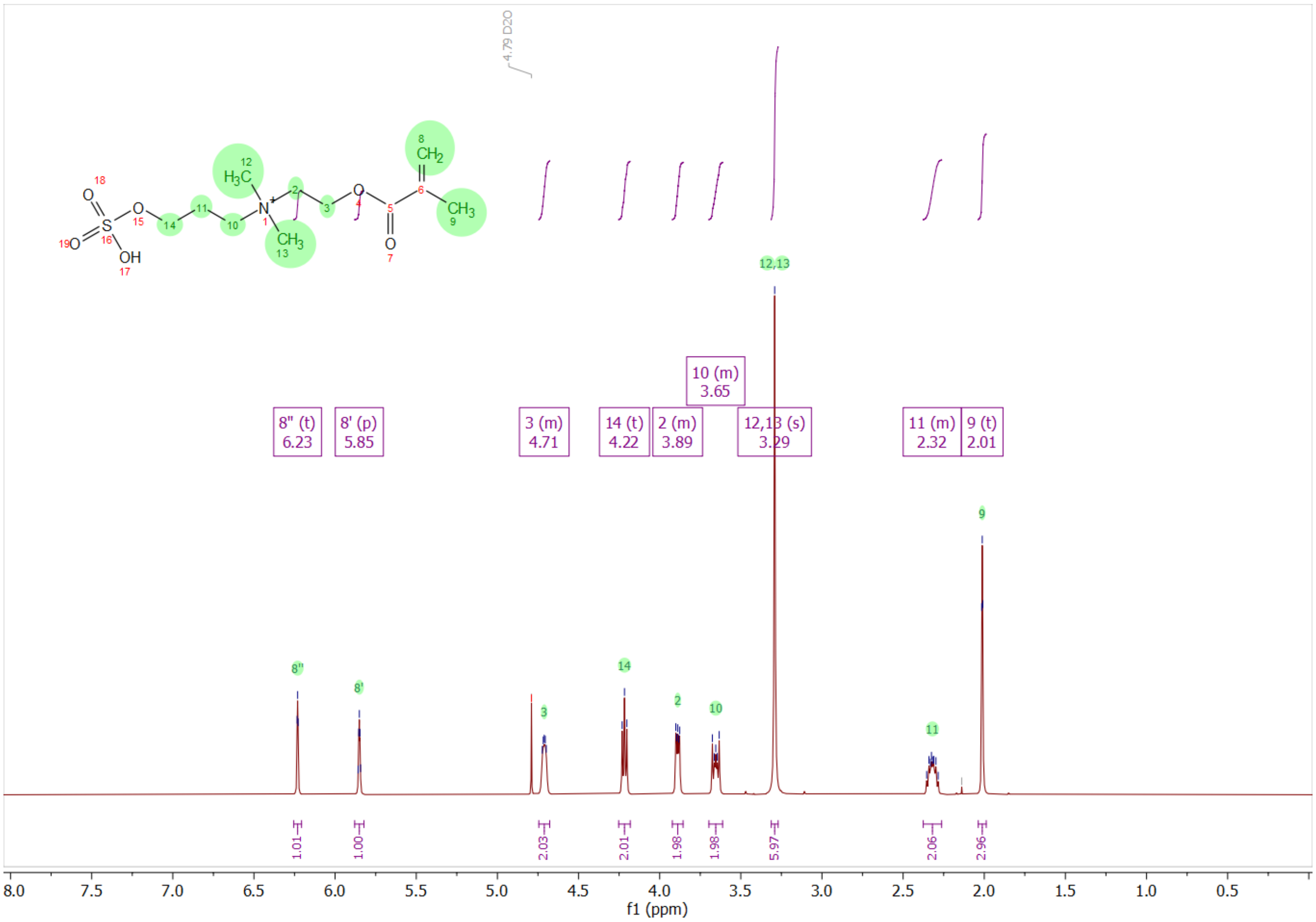

Figure S1. ^{1}H NMR spectrum of ZB monomer.

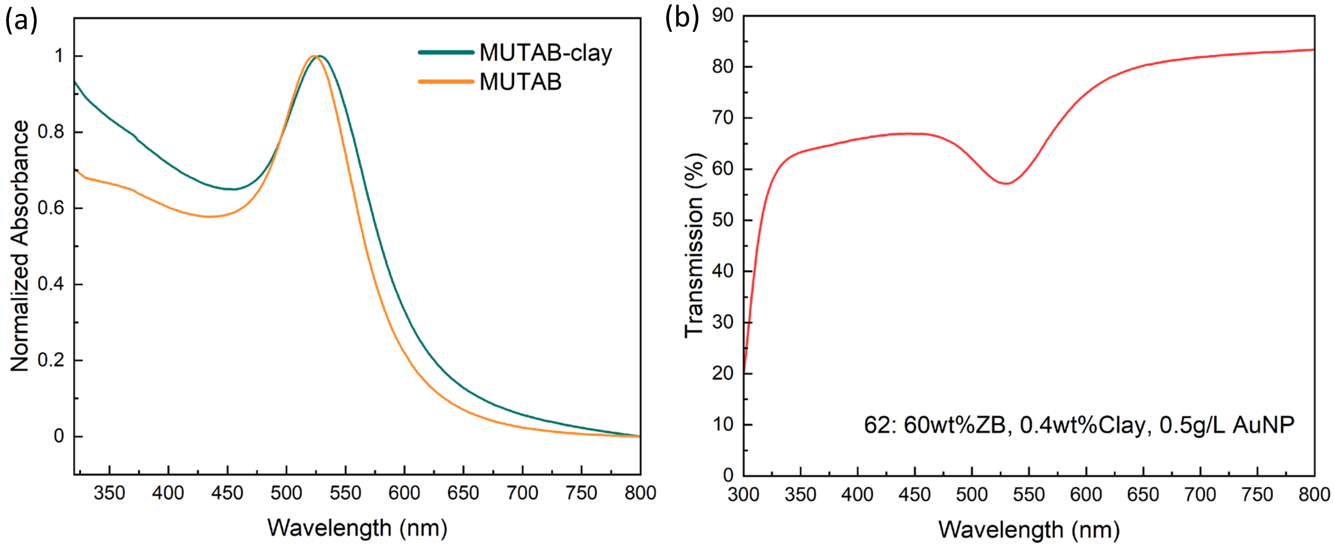

Figure S2. UV-Vis absorbance spectra of cationic AuNP in MQ solution and in presence of nanoclay (a). b) Full spectrum of a hydrogel with incorporated AuNPs.

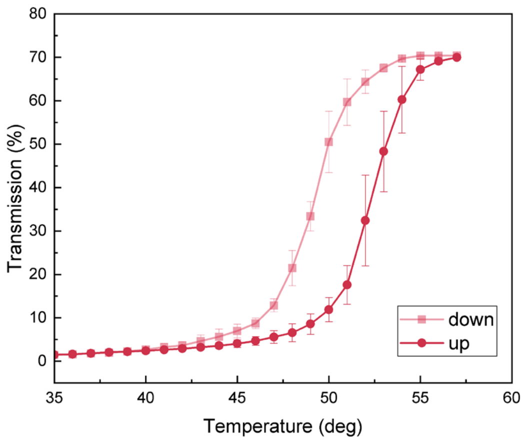

Figure S3. UV-Vis analysis of the phase transition behavior of ZB_{60}-$clay_{1.6}$-AuNP pre-conditioned at 56°C.
